# EHMTI-0143. The association between stress and headache: a longitudinal population-based study

**DOI:** 10.1186/1129-2377-15-S1-F23

**Published:** 2014-09-18

**Authors:** S Schramm, S Moebus, N Lehmann, U Galli, M Obermann, E Bock, MS Yoon, HC Diener, Z Katsarava

**Affiliations:** 1 Institute for Medical Informatics Biometry and Epidemiology, University Hospital, Essen, Germany; 2 Department of Psychology, Center for Psychotherapy University, Zurich, Switzerland; 3 Department of Neurology, University Hospital, Essen, Germany; 4 Department of Neurology, St. Joseph Hospital Ruhr-University, Bochum, Germany; 5 Department of Neurology, Evangelisches Krankenhaus, Unna, Germany

## Introduction

Stress as a trigger for headache is often reported by patients.

## Aims

We studied the association between stress intensity and headache frequency for tension type headache (TTH), migraine and migraine with coexisting TTH (MigTTH).

## Methods

The German Headache Consortium studied a population-based sample of 5,159 participants (21-71years) who were asked every three months between March 2010 and April 2012 about headache and stress. Log-linear regression in the framework of Generalized Estimating Equations was used to estimate regression coefficients presented as percent changes to describe the association between stress intensity (visual analogue scale [VAS] from 0-100) and headache frequency (days/month) stratified by headache subtypes and age groups. Percent changes were adjusted for sex, age, frequent intake of acute pain drugs, drinking, smoking, body mass index and education.

## Results

TTH was reported in 31% participants (48.1±12.5years, 51.5% women, 2.2±3.9 mean headache days/month, 52.3±26.7 mean stress), migraine in 14% (44.8±11.3years, 73.3%, 4.5±5.2days/month, 62.4±23.3), MigTTH in 10.6% (43.5±11.5years, 61.0%, 3.6±4.8days/month, 58.6±24.1), 23.6% of respondents were unclassifiable, 20.8% had no headache. In participants with TTH an increase of 10 points on VAS was associated with an increase of headaches days/month of 6.0% (fully adjusted). Higher effects were observed in younger age-groups (21-30/31-40/41-50/51-60/61-71years: 9.8/10.2/7.0/6.5/3.5%). Similar effects, though slightly lower were observed for migraine (4.3%, 8.1/5.1/3.4/6.3/0.3%) and MigTTH (4.2%, 5.5/6.8/6.9/5.8/-0.7%).

## Conclusions

Our study provides evidence for an association between stress intensity and headache frequency. Our findings are clinically important explaining that patients might benefit from psychological interventions for stress. The benefit might be higher in younger headache sufferers.

No conflict of interest.

